# Fetal heart rate spectral analysis in raw signals and PRSA-derived curve: normal and pathological fetuses discrimination

**DOI:** 10.1007/s11517-023-02953-5

**Published:** 2023-10-27

**Authors:** Giulio Steyde, Edoardo Spairani, Giovanni Magenes, Maria G. Signorini

**Affiliations:** 1 Department of Electronics, Information and Bioengineering, Politecnico di Milano, Piazza Leonardo da Vinci 32, 20133 Milano, Italy; 2 Electrical, Computer and Biomedical Engineering Department, Università di Pavia, 27100 Pavia, Italy

**Keywords:** Electronic fetal monitoring, Cardiotocography, Spectral analysis, Phase-rectified signal averaging

## Abstract

**Abstract:**

Cardiotocography (CTG) is the most common technique for electronic fetal monitoring and consists of the simultaneous recording of fetal heart rate (FHR) and uterine contractions. In analogy with the adult case, spectral analysis of the FHR signal can be used to assess the functionality of the autonomic nervous system. To do so, several methods can be employed, each of which has its strengths and limitations. This paper aims at performing a methodological investigation on FHR spectral analysis adopting 4 different spectrum estimators and a novel PRSA-based spectral method. The performances have been evaluated in terms of the ability of the various methods to detect changes in the FHR in two common pregnancy complications: intrauterine growth restriction (IUGR) and gestational diabetes. A balanced dataset containing 2178 recordings distributed between the 32nd and 38th week of gestation was used. The results show that the spectral method derived from the PRSA better differentiates high-risk pregnancies vs. controls compared to the others. Specifically, it more robustly detects an increase in power percentage within the movement frequency band and a decrease in high frequency between pregnancies at high risk in comparison to those at low risk.

**Graphical abstract:**

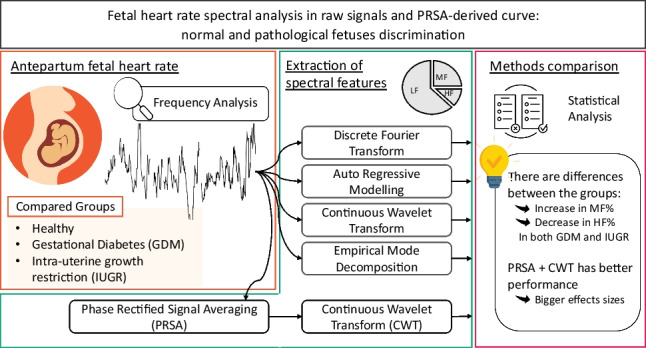

## Introduction

The fetal heart rate (FHR) is a readily available source of physiological information which can be acquired non-invasively either using cardiotocography (CTG) [1] or from the fetal ECG measured through abdominal electrodes (antepartum) [2] or directly on the fetal scalp (intrapartum).

Thanks to its relative ease of acquisition and ability to reflect fetal wellness, electronic FHR monitoring is the most common technique for assessing fetal well-being in pregnancy. An improvement to the FHR analysis came when several biomarkers were proposed in the literature together with systems for the computerized analysis of the signals. This has allowed to overcome some of the limitations linked to simple eye inspection of the traces [3, 4].

In fact, quantitative analysis of the FHR signal allows the identification of important characteristics that can go missed by visual inspection and ensures the reproducibility of the analysis. Moreover, parameters can be used to build classifiers of FHR recording by means of machine learning techniques [5–7].

Despite the advancements made in the last years, however, biomarkers of the most common fetal pathologies, e.g., intra-uterine growth restriction (IUGR), still have limited reliability [8, 9] and most have been tested on a very limited number of subjects.

The rationale behind the study of the FHR antepartum is that disturbances in the normal intrauterine development lead to changes in the autonomic functions that are observable from the cardiovascular regulation [10]. Monitoring these changes is important both in perinatal medicine for their prognostic and diagnostic value and in the framework of the “developmental origin of health and disease” [11] for the possibility to predict disturbances later in life. Among the parameters presented in the literature, those that allow quantifying the frequency distribution of the oscillations in the heart rate variability (HRV) have the desirable property of being physiologically interpretable, since it has been shown that the sympathetic and parasympathetic branches of the autonomic nervous system (ANS) influence the HRV at different frequencies [12].

When analyzing the power spectral density (PSD) of the FHR signal, three main bands are typically defined in analogy with those employed for adults [13] and specifically adapted to the fetal case. These include the very low frequency (VLF), low frequency (LF), and high frequency (HF) bands. In addition, some authors also consider an additional frequency band, called movement frequency (MF), between LF and HF [4]. The exact frequency ranges vary between different authors and have been summarized in [14]. In general, the power in VLF is related to long period and non-linear contributions and gross body movements, LF with mainly sympathetic activity and HF with parasympathetic activity and fetal breathing [4]. MF has been hypothesized to be related to fetal movements and maternal breathing [4] but overlaps with what other authors consider HF [14].

The FHR signal presents characteristics that complicate its analysis in the frequency domain. The variability of the FHR and its frequency distribution change over time. While in adults, the experimental conditions can be easily controlled (for example, asking the subject not to move and breathe at a controlled rate); this is impossible to do in the fetal case, due to frequent changes in behavioral states [15]. It results that the FHR signal is inherently non-stationary, which must be addressed in frequency analysis. Moreover, it is expected that some oscillations in the FHR, like the ones induced by respiratory movements, are transient and possibly not phase-synchronized, which means they may not be captured by standard spectral analysis. Lastly, FHR traces are often very noisy and subject to signal loss.

Since the task of analyzing the frequency content of the FHR signal is not trivial, several methodologies have been applied [16, 17]. In particular, we expect that some of these may be more effective in detecting oscillations even when the assumptions of classical spectral analysis do not hold (more noticeably, stationarity, and linearity). These techniques can be broadly classified as parametric (typically autoregressive (AR)) and non-parametric (based on the discrete Fourier transform (DFT) or the Hilbert–Huang transform) and may use explicit windowing (such as the short-time Fourier transform (STFT)) or not (such as the continuous wavelet transform (CWT)).

More recently, the phase-rectified signal averaging (PRSA) technique has been proposed to detect quasi-periodicities in non-stationary signals [18]. The PRSA is not a method for spectral analysis itself, but rather a technique that produces a compressed version of the original signal (i.e., the PRSA curve) in which the noise is smoothed-out and (quasi) oscillations are highlighted, even in the presence of phase-resetting.

Several works employing measures extracted using PRSA have been applied to FHR analysis: in particular, acceleration and deceleration capacity [19–21], acceleration and deceleration phase-rectified slope [22], and deceleration reserve [23]. Some authors postulate that the deceleration capacity is a measure of the vagal control of the heart rate and the acceleration capacity is a measure of sympathetic activity [24]. However, this assumption has been challenged in [25] and [23].

In this study, we propose a different approach for the analysis of the PRSA curve derived from FHR signals. Similarly to what proposed by Bauer et al. [18], we perform the CWT of the PRSA curve but evaluate the relative distribution of the oscillations in the frequency domain instead of evaluating it only at specific scales, like it is done when calculating acceleration and deceleration capacity. This allows to compare the proposed method with classical spectral analysis and permits to rely on the frequency content of the PRSA curve to investigate the activity of the ANS, even if indirectly.

The proposed approach is compared to four more traditional methods used to perform spectral analysis of FHR signals from cardiotocographic recordings [17], i.e., DFT, AR modeling, CWT, and empirical mode decomposition (EMD).

The paper describes the performances of the proposed method and of the other existing in the detection of changes in the FHR due to two very common complications of pregnancy, which are expected to produce changes in the FHR signal characteristics: intrauterine growth restriction (IUGR) and gestational diabetes (GDM). For the analyses, we used a very large database of antepartum CTG recordings collected at different gestational ages.

## Methods

### Dataset description

The dataset employed is presented in more detail in [26]. It currently contains a total of 24492 cardiotocographic recordings collected and annotated at Federico II University Hospital in Naples, Italy. Data were collected in accordance with the Declaration of Helsinki after approval of the local Ethics Committee. All subjects included in the dataset signed an informed consent, and data were completely anonymized before the analysis by our clinical partners.

The FHR signal is sampled at 2Hz with a resolution of 0.25 bpm. This sampling frequency is the one used by the 2CTG2 software [27] and was chosen because it is a reasonable compromise to achieve enough bandwidth and an acceptable accuracy of the FHR signal. Indeed, it allows to correctly represent all the frequencies contained in the FHR signal estimated by the cardiotocograph, while minimizing the number of repeated samples. It should be noted, in fact, that commercial ultrasound cardiotocographs provide an approximation of the true FHR which is low-passed by the smoothing effect produced by the autocorrelation procedure embedded in the firmware. Moreover, reading the value of FHR at higher sampling frequencies (e.g., 4Hz) produces non-negligible distortions in the power spectrum of the signal, since cardiotocographs replicate the current values until a new one is detected.

CTG signals are often affected by artifacts, signal loss, and noise. A quality index (i.e., good, acceptable, interpolated) provided by the cardiotocograph is also available for each data point of the signals. The length of each recording varies between 20 min and 1 h according to the clinician’s decision. Along with the FHR, each recording includes the uterine contractions’ signal, the fetal movement (FM) series (which is set to 1 when the mother presses a button and 0 when she does not), and annotations by the clinician noting any known maternal and fetal pathologies.

In this study, we selected the first 20 min of recordings that have no more than 10% of interpolated points in this window and were performed between the 32nd and 38th gestational week. The choice of using only the first 20 min of each recording was made to avoid possible biases that may arise from the use of series of different lengths, since recordings belonging to complicated pregnancies tend to be longer. We then identified three distinct populations: controls, i.e., physiological pregnancies without known maternal or fetal pathologies, IUGRs (diagnosed when the fetal weight is lower than the 10th percentile for gestational age and are present alterations in the umbilical artery flow, in agreement with [28]), and GDMs (diagnosed following a positive 1-step glucose tolerance test [29]). For each week, we selected a subset of equal size from each population randomly downsampling from the largest groups. The final dataset thus contains the same numerosity for the three analyzed groups in each week and includes a total of 2178 recordings.

Recordings were then divided into two groups: from week 32 of gestation to week 36 (pre-term) and from 37 to 38 (early-term), since considerable differences in fetal maturation are expected between the two periods. The first includes a total of 1161 recordings and the second 1017.

### Time-frequency analysis: classical methods

Among the traditional methods to perform spectral analysis we selected: DFT, AR modeling, CWT, and EMD. All these methods aim to estimate the PSD, albeit in very different ways. In this paragraph, we briefly outline some details about their implementation and their differences.

#### Explicit windowing: DFT and AR

The DFT and AR are the most common methodologies to estimate the PSD. Since both make the assumption of stationarity, the FHR signal was divided into windows of 2 min overlapped by 1 min, which is a compromise between spectral resolution and the fulfillment of the stationarity condition. Inside each window, we removed the linear trend from the signal. The DFT was estimated by applying the fast Fourier transform and directly used to estimate the PSD. Parametric spectral analysis with AR models was performed as reported in [4]. The order was set to 12, and the parameters were estimated using the Levinson–Durbin algorithm.

#### Time-varying algorithms: CWT and EMD

Time-varying algorithms, i.e., the CWT and the EMD, are expected to provide better time-frequency resolution compared to methods that employ explicit windowing. Moreover, since the EMD assumes neither stationarity nor linearity of the signal [30], it appears to be particularly suitable for the analysis of the FHR. Prior to their application, the signal was detrended by removing the moving average computed over windows of 1 min and padded with a periodized extension of 240 samples to reduce distortions at the borders.

For the computation of the CWT, we employed the Morlet wavelet with non-dimensional central frequency equal to 6. The scales “s” were defined according to (1).

1 $$\left\{\begin{array}{c}{\delta}_j={J}^{-1}\bullet {\log}_2\left(\frac{N\bullet {\delta}_t}{s_0}\right)\\ {}{s}_j={s}_0\bullet {2}^{j{\delta}_j},\kern0.5em j=0,1,\dots, J\end{array}\right.$$

*N* is the length of the signal and δ_*t*_ the sampling period (i.e., 0.5s). *s*_0_ was set to 1s ($2 \cdot{\delta}_t$) and J to 179. Wavelet software was provided by C. Torrence and G. Compo [31] and is available at the URL: http://paos.colorado.edu/research/wavelets/.

The EMD was computed using the Complete Ensemble Empirical Mode Decomposition with Adaptive Noise (CEEMDAN) algorithm, which is more robust to noise [32]. The number of realizations was set to 30, the noise standard deviation to 0.2 times the standard deviations of the signal, and the maximum number of shifting iterations to 50. The SNR increases for every stage.

#### Feature computation

Spectral features, i.e., LF%, MF%, and HF%, are computed by integrating the spectra (or spectrograms) over the frequency and considering the mean over time. Segments with more than 5% of interpolated points were excluded from averaging. In the present paper, we consider the following frequency bands: LF (0.03–0.15 Hz), MF (0.15–0.5 Hz), and HF (0.5–1 Hz), following the definition provided in [4]. Spectral features are expressed as percentages of the total power. Indeed, what we are interested in quantifying in this study is the frequency distribution of the signal variability, rather than the variability itself. We do not report the values of VLF, which can be trivially obtained from the others.

### PRSA spectrum evaluation

In this section, we briefly describe the PRSA technique, which was introduced by Bauer et al. [18]; the computation of the PRSA spectrum [33]; and the proposed approach for its evaluation and extraction of spectral features.

#### The PRSA curve

**Fig. 1:**
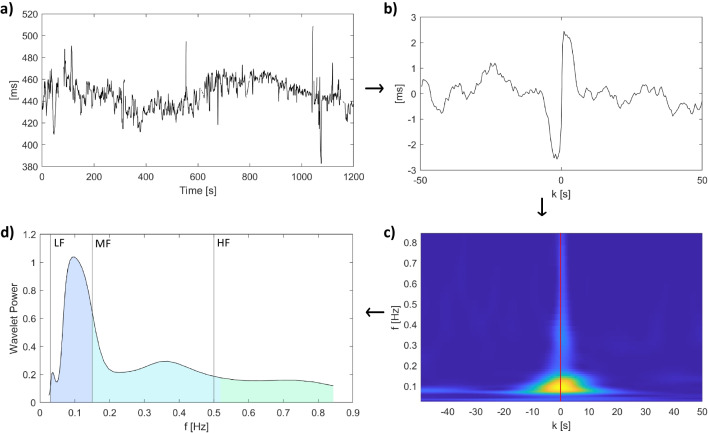
Illustration of the technique employed to compute the PRSA spectrum. **a** fHR signal; **b** PRSA curve; **c** CWT spectrogram of the PRSA curve obtained by squaring wavelet coefficients; **d** CWT spectrum at *k*=0 (section of the spectrogram)

Deceleration anchor points (*x*_*dec*_) are defined as samples that satisfy the condition:

2 $$\frac{1}{T}\sum\nolimits_{i=0}^{T-1}x\left[t+i\right]>\frac{1}{T}\sum\nolimits_{i=1}^Tx\left[t-i\right]$$

where *x* is the FHR series expressed in milliseconds (Fig. 1a). For each *x*_*dec*_, a window of length 2L is defined taking the values of the original signal that go from *x*_*dec* − *L*_ to *x*_*dec* + *L* − 1_. The PRSA curve (${x}_k^{PRSA}$) is constructed by averaging all these windows (Fig. 1b). This procedure highlights components that are phase-synchronized with the anchor points and cancels out the others.

In this study, T was set to 1 sample (i.e., 0.5 s). We acknowledge that the choice of such a small value of T can make the procedure more sensitive to noise, but selecting a bigger value would filter the power in the HF band, which is of interest in this study. Indeed, the larger the value of T, the lower the frequency of the components that are highlighted by the PRSA [34]. To reduce the influence of noise, signal samples with poor quality were prevented from being anchor points and were not included in the averaging procedure. L was set to 100 samples.

#### The PRSA spectrum

To compute the PRSA spectrum (PRSA_Spt), we first computed the scalogram ${X}_w^{PRSA}$ using the CWT according to Eq. 3 in a similar fashion to [33]

3 $${X}_w^{PRSA}\left(s,p\right)={\sum}_{k=-L}^{L-1}{x}_k^{PRSA}\bullet \frac{w\left[\left(k-p\right)/s\right]}{s}$$

*s* is the scale, *p* is the position, and *w* is the mother wavelet. We employed the analytic Morse wavelet with γ equal to 3 and time-bandwidth product equal to 60 and applied L1 normalization. The spectrogram is obtained as the square of the wavelet coefficients (Fig. 1c) and is evaluated at *k*=0 (Fig. 1d), thus obtaining a single spectrum, i.e., PRSA_Spt.

As discussed in [18], the PRSA_Spt presents relevant differences compared with conventional spectral analysis. The signal-to-noise ratio is improved by two effects:

- Short patches of periodicities with a particular frequency that are not phase synchronized cancel out in conventional spectral analysis, while, due to the way it is defined, they all contribute to the PRSA curve and, therefore, to its spectrum. On the other hand, non-periodic components are smoothed-out by the averaging procedure applied by the PRSA.
- A sinusoidal component of amplitude *A*_*f*_ produces an oscillation proportional to ${A}_f^2\bullet f$ in the PRSA curve. It derives that while its contribution in the conventional power spectrum is proportional to ${A}_f^2$ in the PRSA_Spt is proportional to ${A}_f^4\bullet {f}^2$. Therefore, a 1/f noise has an approximately flat PRSA spectrum. This makes it substantially easier to identify deviations from the standard scaling behavior of long-term correlated series caused by quasi-periodicities.

Both these properties are useful in the analysis of FHR signal. Indeed, oscillatory patterns in the FHR are usually transient and not phase synchronized. Moreover, most of the variability is contained at very-low frequencies, consistently with a long-term correlated series, rendering it difficult to identify superimposed quasi-oscillations, especially at high-frequency that, despite their smaller amplitude, may offer important information on fetal physiology.

#### PRSA-derived spectral features

The method we present in this study is a variation of the method presented in [33] to evaluate the PRSA_Spt. Here, instead of considering the Wavelet coefficients at a single scale, we propose to quantify the distribution of the oscillations integrating the PRSA_Spt (Fig. 1d) in the frequency bands used in traditional spectral analysis (i.e., LF MF and HF). The features considered are ultimately the percentages of power obtained integrating the PRSA_Spt in the frequency bands described previously.

It should be noted that spectral features computed after the application of PRSA can no longer be regarded as an estimate of the distribution of the signal variance in the frequency domain. Rather, it is a measure of the localization in frequency of the oscillations that survive the PRSA procedure evaluated in descending signal segments.

This approach allows using the distribution in the frequency domain of the oscillations to estimate the fetal autonomic activity, rather than the fact that they are aligned around portions in which the signal increases or decreases. Therefore, unlike the other features that can be extracted from the PRSA, the ones proposed should not suffer from the limitations in the interpretation of their physiological meaning which have been pointed out in [25] and [23].

### Statistical methods

Since the features discussed are not normally distributed, we employed the Mann-Whitney *U* test to evaluate differences between the two high-risk groups and controls and computed the Cohen’s *r* size effect to quantify them [35]. Results are considered significant when *p*<0.0167, following Bonferroni correction for multiple comparisons. Confidence intervals were computed using empirical bootstrap with 1000 repetitions.

All analyses were conducted using MATLAB R2022a.

## Results

Table 1 reports values in relative spectral power obtained in the first group of weeks and Table 2 in the second. It can be noticed that the values obtained from classical methods of spectral analysis did not show substantial differences among each other. On the other hand, the PRSA_Spt accentuates higher frequency components, a result consistent with the scaling behavior of the PRSA_Spt described in paragraph 2.3. As a result, the PRSA_Spt reports higher values for MF% and HF% compared to the other methods. Moreover, employing classical spectral analysis a considerable portion of the total power is contained in the VLF band (i.e., f<0.03 Hz), while the power in this band is almost entirely filtered-out by the PRSA.

**Table 1:** Medians and quartiles of the spectral features at weeks 32–36 obtained with the methods discussed. All values are reported in percentages of the total power

	LF%			MF%			HF%		
	Control	GDM	IUGR	Control	GDM	IUGR	Control	GDM	IUGR
DFT	39 (33–45)	39 (33–46)	37 (32–44)	5.6 (4.0–7.0)	6.4 (4.5–8.7)	6.0 (4.5–7.9)	1.8 (1.2–2.7)	1.7 (1.1–2.5)	1.5 (1.1–2.4)
AR	35 (25–48)	35 (25–48)	32 (25–43)	4.3 (3.0–5.8)	5.2 (3.5–7.1)	5.0 (3.6–6.7)	1.8 (1.2–2.8)	1.9 (1.2–2.8)	1.5 (1.1–2.4)
CTW	40 (34–46)	41 (35–46)	40 (35–46)	5.3 (3.9–7.2)	6.5 (4.5–8.7)	6.3 (4.6–8.7)	1.7 (1.1–2.4)	1.5 (1.1–2.4)	1.4 (1–2.3)
EMD	33 (28–39)	34 (29–40)	33 (28–39)	5.5 (4.2–7.1)	6.6 (5.0–9.1)	6.4 (4.9–8.3)	3.2 (2.1–4.7)	3.0 (1.9–4.5)	2.7 (1.9–4.6)
PRSA_Spt	38 (25–52)	36 (22–50)	37 (23–50)	20 (16–25)	30 (22–37)	28 (20–35)	33 (19–46)	25 (17–37)	25 (14–38)

**Table 2:** Medians and quartiles of the spectral features at weeks 37–38 expressed as percentages of the total power

	LF%			MF%			HF%﻿		
	Control	GDM	IUGR	Control	GDM	IUGR	Control	GDM	IUGR
DFT	37 (32–43)	38 (33–44)	37 (32–44)	6.0 (4.5–7.8)	6.3 (4.7–8.1)	5.9 (4.7–8.2)	1.7 (1.1–2.5)	1.7 (1.2–2.5)	1.6 (1.1–2.3)
AR	32 (22–41)	33 (25–44)	33 (24–43)	4.7 (3.4–6.4)	5.1 (3.6–7.0)	4.9 (3.5–4.8)	1.7 (1.1–2.5)	1.6 (1.2–2.4)	1.6 (1.1–2.3)
CTW	38 (33–44)	39 (35–45)	39 (34–46)	5.9 (4.4–7.9)	6.5 (4.5–8.5)	6.1 (4.7–8.5)	1.4 (0.9–2.3)	1.5 (0.9–2.3)	1.5 (1.0–2.1)
EMD	32 (27–38)	33 (28–39)	33 (28–28)	6.4 (4.9–8.5)	6.6 (4.9–9.0)	6.6 (4.9–9.0)	2.8 (1.9–4.6)	2.9 (1.9–4.5)	2.8 (1.9–4.1)
PRSA_Spt	38 (24–53)	37 (24–53)	36 (26–51)	25 (19–33)	29 (22–37)	28 (21–36)	26 (14–41)	25 (14–36)	23 (14–36)

**Fig. 2:**
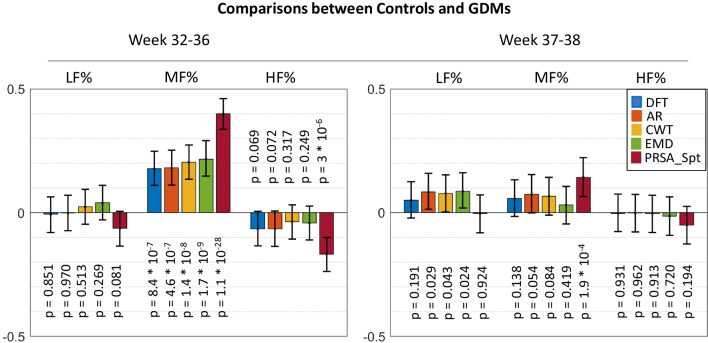
Cohen’s effects size for the Mann-Whitney *U* test in comparison between controls and GDMs. Positive values indicate an increase in the high-risk population. Confidence intervals are computed using empirical bootstrap with 1000 repetitions

**Fig. 3:**
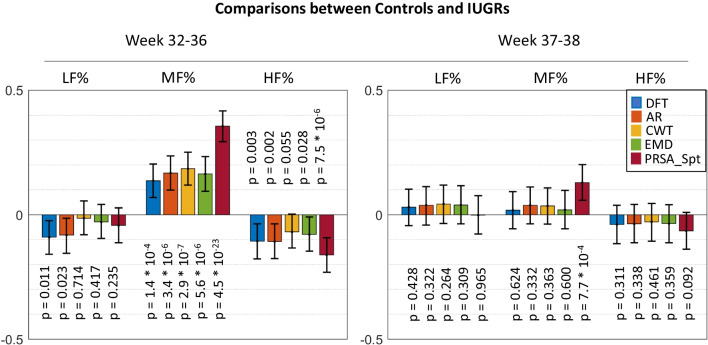
Cohen’s size effects for the Mann-Whitney *U* test in comparison between controls and IUGRs and *p*-values

The results related to the comparison between GDMs and controls are reported in Fig. 2, while comparisons between IUGRs and controls in Fig. 3.

In general, more differences were identified in the first group of weeks. This result is in agreement with other studies [5, 36], which also found more differences in the pre-term period compared to the early-term, even though the FHR features analyzed were different.

In the first groups of weeks, all methods identified a significant increase in MF% both in the comparison between GDMs against controls and IUGRs against controls. It can be noticed that the PRSA_Spt method resulted in a significantly bigger size effect. Indeed, traditional methods resulted in both cases in small size effects (lower than 0.2), while the ones obtained with PRSA_Spt were moderate (0.40 for GDMs and 0.36 for IUGRs).

Classical methods did not detect any significant difference in the HF band for GDMs, while a small reduction was identified by AR and DFT in the IUGR population. The PRSA_Spt instead evidenced significantly lower values in the HF band for both pathological groups compared to controls. The effect sizes however are small (−0.17 for GDMs and −0.16 for IUGRs), even though the *p*-values are well below the 5% significance level (3×10^−6^ and 7.5×10^−6^ , respectively).

No differences were identified in the LF band for GDMs, while a small reduction was identified by AR in the GDMs. The PRSA_Spt did not evidence a significant difference in this band.

In the second group of weeks, some traditional methods, but not PRSA_Spt, suggest a slight increase in LF% for GDMs. An increase in MF% in both high-risk groups was identified only by PRSA_Spt. No differences were identified in HF%.

**Fig. 4:**
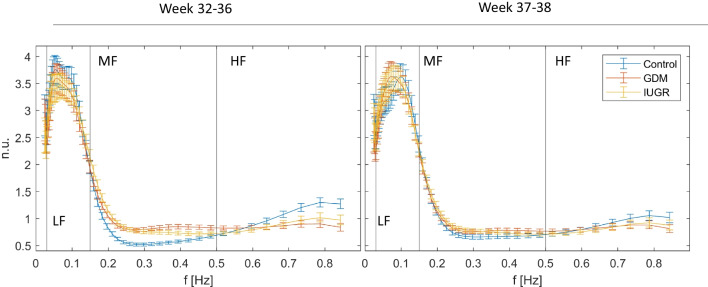
Average normalized deceleration-related PRSA_Spt with 95% confidence intervals computed using empirical bootstrap with 1000 repetitions. Notice that high-risk pregnancies show similar behaviors (increase in MF and decrease in HF) and more relevant differences are observed in the first group of weeks. Spectra have been normalized by dividing by the total power of the PRSA_Spt and are presented in normalized units

The differences between groups identified with the PRSA_Spt method can also be clearly seen by looking at the average spectra, which are reported in Fig. 4. It can be noticed that at weeks 32 to 36 high-risk pregnancies and controls show different behaviors in MF and HF, while they substantially overlap in the LF band. Moreover, it is clearly visible a reversal of the trends of physiological and high-risk pregnancies around 0.55Hz. The differences among the average PRSA_Spt for the three analyzed groups at weeks 37 and 38 are much less evident.

As reported in Table 3 the FHR variance, or total power, shows a trend which aligns with expectations, i.e., higher variability in controls. However, the differences are not statistically significant.

**Table 3:** Medians and quartiles of the total power of the fHR

	32–36			37–38		
	Control	GDM	IUGR	Control	GDM	IUGR
Tot. Pow. [ms^2^]	259 (158–404)	220 (143–391)	243 (137–391)	297 (178–452)	265 (166–418)	266 (172–451)

## Discussion

The purpose of the research work presented in this manuscript is twofold. Firstly, we elaborated on the applicability and robustness of spectral analysis in FHR signals using a very large dataset of healthy and complicated pregnancies and compared several existing methods of spectral analysis. Secondly, we proposed a different approach for evaluating the spectrum of the PRSA and compared the results with those obtained with more classical spectral analysis methods.

We found that the application of the PRSA method before computing the spectra improves substantially the capability to distinguish between uncomplicated and high-risk pregnancies compared to classical spectral analysis. On the other hand, we did not observe substantial differences among the other methods tested.

Classical methods for time-frequency analysis have the advantage that allows localizing in time the results, allowing, for example, to investigate how the fetus reacts to stimuli (e.g., uterine contractions [37]) and differentiating among different fetal behavioral states [38]. Moreover, standard spectral analysis has a direct physiological interpretation. In fact, changes in the PSD of the HRV signal have been connected to the action of regulatory mechanisms of the autonomic nervous system [12].

Concerning the PRSA method, our results suggest that it presents a marked advantage in the discrimination abilities, which could lead to an advantage in clinical practice, at the expenses of providing a less direct physiological interpretation of the results. Indeed, it does not directly measure the oscillations in the signal as in classical spectral analysis, but only the ones that survive the averaging procedure.

We attribute the better ability of the PRSA to discriminate among complicated and uncomplicated pregnancies to its intrinsic reduce insensitivity to noise and capability of capture oscillations that are not phase synchronized. Indeed, the averaging procedure that is applied when computing the PRSA curve cancels-out non periodic components. On the other hand, periodic components that are not phase-synchronized (“phase jumps,” as reported by Bauer et al. [18]) are enhanced by the procedure. We speculate that in the FHR these types of short and not phase-synchronized oscillations may be induced by transient events, like fetal movements of the body and fetal respiratory movements. Moreover, the intrinsic non-stationarity and non-regularity of the FHR signal calls for the application of a method which is robust to noise, as the PRSA method has demonstrated to be. Lastly, as demonstrated in [18], the PRSA method is advantageous for the detection of quasi-oscillations superimposed on a long-term correlated series, as it is the case of FHR. While traditional spectral analysis may be unable to disentangle these oscillations, the PRSA is able to reliably detect them.

The most relevant difference observed between controls and high-risk groups is a relative increase in the power in MF for the latter. Previous studies have attributed oscillations in this band to the presence of fetal movements [4]. However, we did not observe a significant increase in their occurrence as perceived by the mother in the high-risk groups. This may be indicative that other pathophysiological mechanisms are at the source of this pattern. Indeed, this frequency band is arguably the one with the least clear physiological interpretation, which requires further study. Quite interestingly, in [38], the authors found very little power in this band in uncomplicated low-risk pregnancies.

The reduction in HF that was observed in the pathological groups is consistent with a reduction of respiratory movements. Concerning IUGR fetuses, this is backed up by other studies which found that respiratory movements are lower in speed, power, and intensity and in general have lower “quality” [39]. We did not observe significant differences between the groups in LF% with PRSA_Spt, while minimal differences were identified using other methods. This is probably due to the scaling behavior of the PRSA and the choice of *T* = 1. Indeed, quasi-oscillations that are enhanced the most lay around 1/2.5*T* [18] which in our case corresponds to the HF band. We did identify a significant reduction in LF% in the high-risk groups at weeks 32–36, but not at weeks 37–38 using *T* = 2 (size effect: −0.19 for GDM and −0.15 for IUGRs). This, however, came at the expense of substantially reduced differences in MF% and HF%. At *T* = 4, the relative power in HF was virtually null.

Interestingly, we did not find substantial differences when considering the acceleration-related PRSA curve instead of the deceleration-related one.

Ultimately, the results show that spectral analysis of the FHR can be a useful tool to distinguish complicated and uncomplicated pregnancies, which is made substantially more robust by the use of the PRSA. This may have important clinical applications. Concerning IUGR identification, the use of FHR as a biomarker for screening of the pathology may be useful, especially in low-income countries. Indeed, low-cost and easy to use instrumentation for FHR monitoring is already available on the market. However, reliable IUGR detection via FHR only is still challenging, hence the need to introduce more reliable and robust features. Moreover, FHR analysis may be useful to differentiate between real IUGR and SGA fetuses that can be confused using only echography.

Regarding diabetes, pregnancy management in presence of the pathology is still highly uncertain, and developing methods able to provide additional information to the clinicians may be useful for optimal management. Indeed, identifying variations induced by the pathology in the FHR may be a first step towards a better understanding of the effect of maternal diabetes on fetal development and hopefully be useful for risk stratification in future studies.

One limitation of the present study is that we only analyzed data collected using CTG with a sampling frequency of 2Hz. We acknowledge that the results may change using FHR acquired with different techniques, such as fetal electrocardiography (fECG). Indeed, high-resolution fECG recordings allow to obtain the true beat-to-beat fetal heart rate, which is recommended for PRSA analysis [33]. Moreover, high-resolution fECG recordings would allow to better quantify high-frequency components of the FHR signal which, as a result of the application of the autocorrelation technique, are partially filtered-out by ultrasound cardiotocographs. However, fECG is still less commonly used in clinical practice [40] and databases of sufficient size that include both complicated and uncomplicated pregnancies are currently not available.

Numerous cardiotocographs provide the values of FHR every 250 ms (4 Hz). To obtain comparable results in this setting, the values of T and L should be multiplied by 2 (i.e., T=2 and L= 200). It should be noted however that if in the 250 ms time window, there is no detection of a new heartbeat; the previous FHR value is repeated. This procedure causes a frequent repetition of FHR values, which affects the frequency content. To better understand how using a higher sampling frequency affects the results of the PRSA-based methodology, we ran some additional simulation studies, not shown in this manuscript, using surrogate data sampled at 4 Hz. The simulations show that when using higher sampling frequencies results are similar, but the spectral power is slightly lower, especially in the HF band. We speculate that this effect arises from increasing the number of signal samples, which increases the number of windows used in the averaging procedure of the PRSA, since about half of the samples are anchor points, but does not introduce new quasi-oscillations. Therefore, it produces a PRSA curve with generally lower amplitude, and, as a result, the PRSA_Spt has lower power. This effect is more accentuated in the HF range (over 0.5 Hz), since the new windows will be shifted by approximately ±1 sample, resulting in high frequency components being probably in counterphase. This further underlines the fact that it is important to emphasize that, when analyzing CTG traces, it must be considered that they are an approximation of the true RR series. While a low sampling frequency limits the possibility to observe higher frequency components, increasing the sampling frequency generates spurious samples which may impair the quality of the spectral estimation.

Moreover, we selected 20 min from each recording, which is the minimum length available for all. This is a relatively short signal length for the application of the PRSA technique that would benefit from the use of longer series. Indeed, we believe that employing longer signals, such as those that can be obtained by non-invasive fECG, would likely improve the robustness of the results.

Another limitation is that we did not differentiate between behavioral states, which is something we aim to do as future development. Moreover, we hope that, in the future, longitudinal clinical studies will be carried out to test the predictive value of the features discussed for risk stratification, which is the ultimate goal of antepartum FHR analysis.

## Conclusions

The analysis in the frequency domain of the FHR provides useful insights into the fetal physiology, since it allows to assess non-invasively the functioning of the ANS. In this paper, we compared four relatively traditional methods to perform spectral analysis and a novel approach based on the CWT of the PRSA curve and conclude that the latter allows to identify more clearly the differences in the frequency content of the FHR induced by GDM and IUGR. We believe that this approach may have relevant applications, for example, for improving multi-parameters classification and assessment of fetal autonomic activity.
